# Immune checkpoints on T and NK cells in the context of HBV infection: Landscape, pathophysiology and therapeutic exploitation

**DOI:** 10.3389/fimmu.2023.1148111

**Published:** 2023-03-28

**Authors:** Lucile Dumolard, Caroline Aspord, Patrice N. Marche, Zuzana Macek Jilkova

**Affiliations:** ^1^ University Grenoble Alpes, Inserm U 1209, CNRS UMR 5309, Team Epigenetics, Immunity, Metabolism, Cell Signaling & Cancer, Institute for Advanced Biosciences, Grenoble, France; ^2^ R&D Laboratory, Etablissement Français du Sang Auvergne-Rhone-Alpes, Grenoble, France; ^3^ Hepato-Gastroenterology and Digestive Oncology Department, CHU Grenoble Alpes, Grenoble, France

**Keywords:** HBV, T cells, NK cells, immune checkpoint molecules, anti-viral activity

## Abstract

In hepatitis B virus (HBV) infection, the interplay between the virus and the host immune system is crucial in determining the pathogenesis of the disease. Patients who fail to mount a sufficient and sustained anti-viral immune response develop chronic hepatitis B (CHB). T cells and natural killer (NK) cells play decisive role in viral clearance, but they are defective in chronic HBV infection. The activation of immune cells is tightly controlled by a combination of activating and inhibitory receptors, called immune checkpoints (ICs), allowing the maintenance of immune homeostasis. Chronic exposure to viral antigens and the subsequent dysregulation of ICs actively contribute to the exhaustion of effector cells and viral persistence. The present review aims to summarize the function of various ICs and their expression in T lymphocytes and NK cells in the course of HBV infection as well as the use of immunotherapeutic strategies targeting ICs in chronic HBV infection.

## 1 Introduction

Hepatitis B virus (HBV) is a double-stranded DNA virus that infects liver parenchymal cells (i.e., hepatocytes) and is responsible for nearly 1 million deaths annually (1, 2).An effective hepatitis B preventive vaccine has been developed, but 5–10% of vaccinated individuals are non-responders, and vaccination availability remains very low in certain geographical regions (3–5).

In general, acute HBV infection is efficiently controlled by the host immune system and is spontaneously resolved in about 90–95% of adults, while only a minority of infants and young children exhibit this immune capacity (5). Those who fail to achieve immune clearance during first six months after infection develop chronic hepatitis B infection (CHB). Today, around 300 million people are living with CHB, and they are at risk of developing liver complications such as fibrosis, cirrhosis, and hepatocellular carcinoma (HCC) due to sustained liver inflammation (1, 6). Current antiviral therapies for HBV infection rely on nucleoside/nucleotide analogs (NUCs) that are highly efficient at suppressing viral replication. However, they do not completely eradicate the virus (except in a minority of patients), as characterized by the persistence of covalently closed circular DNA (cccDNA) within hepatocytes, and such, long-term treatment must be taken, often for life (7). Therefore, novel therapies to achieve HBV functional cure are needed. Such therapies may require a combination of antiviral and immunomodulatory approaches to reshape a potent antiviral immune response against HBV.

Immune checkpoints (ICs) play key roles in the immune response through the regulation of immune cell functional response by providing either inhibitory or stimulatory signals upon interactions with their ligands. Immune cells naturally express inhibitory ICs to maintain immune tolerance and prevent the immune system’s over-activation, therefore limiting self-damage by controlling the amplitude of the immune response. On the other hand, stimulatory ICs or co-stimulatory molecules are often required to fully activate immune cells and promote an effective immune response. Due to their immune-regulatory properties, ICs have been widely utilized as targets for immunotherapy to enhance host immune responses in patients with cancer as well as in chronic infections such as HBV infection (8).

In this review, we summarize the current knowledge about inhibitory and stimulatory ICs in T cells and NK cells and their role in the pathogenesis of chronic HBV infection, and discuss the relevance of therapeutically targeting ICs for clinical applications.

## 2 Key role of immune checkpoint molecules in the regulation of T cell- and NK cell-mediated anti-viral immune responses

Some ICs are considered inhibitory as they are well-known for negatively regulating T cell functions, such as programmed death protein 1 (PD-1), cytotoxic T lymphocyte antigen-4 (CTLA-4), T cell immunoglobulin domain and mucin domain 3 (TIM-3), lymphocyte activation gene-3 (LAG-3) and T cell immune receptor with immunoglobulin and ITIM domain (TIGIT) and B- and T-lymphocyte attenuator (BTLA). Nonetheless, this vision has been challenged lately as their suppressive effects on immune cells seem context-dependent, especially for NK cells. During T cell activation, after the first signal provided by T-cell receptor (TCR) engagement with major histocompatibility complex (MHC-I or II)/peptide complex, co-stimulatory molecules provide second signals to maximize T cell activation and function (9).

**Figure 1:**
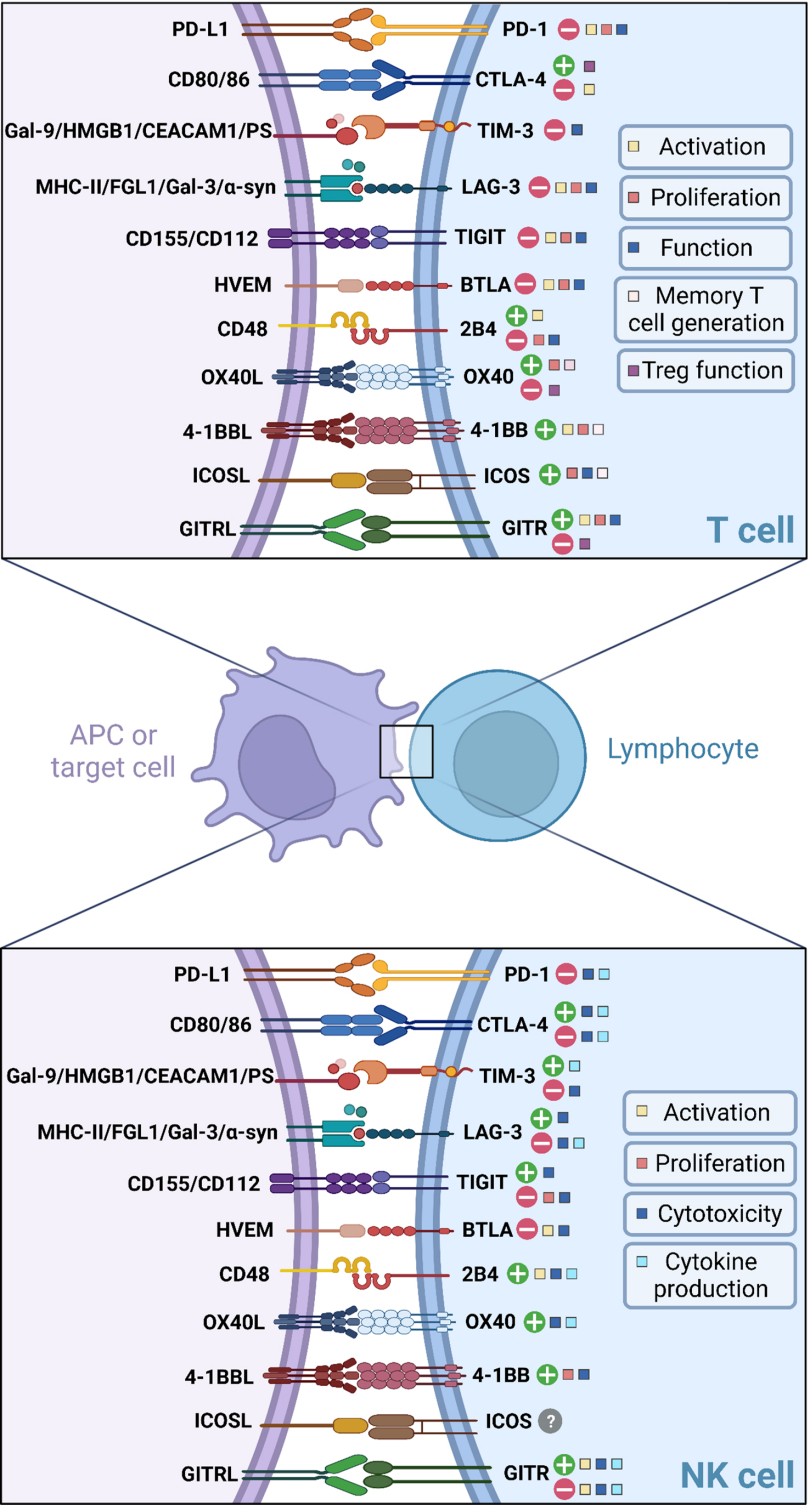
Current knowledge about immune checkpoint signalling in T cells and NK cells.

Besides CD28, other co-stimulatory molecules can efficiently promote T cell activation such as OX40, 4-1BB, inducible T-cell co-stimulator (ICOS) and glucocorticoid-induced tumor necrosis factor-related protein (GITR). Although their stimulatory role in the context of T cell activation has already been demonstrated, their impact on NK cell function remains unclear. The role of these ICs in both T cells and NK cells is summarized in **Figure 1** and **Table 1**.

**Table 1:** ICs and their roles on T cell and NK cell functions.

Immune Checkpoint	Ligands	Impact on T cell function	Impact on NK cell function
PD-1	PD-L1, PD-L2	· Inhibition of T cell activation, proliferation and effector functions (cytotoxicity and cytokine production)	· Inhibition of NK cell effector functions (cytotoxicity and cytokine production)
CTLA-4	B7.1 (CD80), B7.2 (CD86)	· Inhibition of T cell activation · Induction of T cell anergy	· Opposite role documented: activation or inhibition of NK cell effector functions
TIM-3	PS, HMGB1, CEACAM1, Gal-9	· Inhibition of T cell effector functions · Induction of T cell apoptosis	· Different impact: promotion of IFN-γ production or inhibition of NK cell cytotoxicity
LAG-3	MHC class II, FGL1, Gal-3, α-syn	· Inhibition of T cell activation, proliferation and cytokine secretion	· Inhibition of cytokine production · Conflicting results on NK cell cytolytic activities: no modulation, activation or inhibition
TIGIT	CD155 (PVR), CD112 (PVR2)	· Inhibition of T cell activation, proliferation and effector functions	· Inhibition of NK cell proliferation · Conflicting results on NK cell cytolytic activities: no modulation or inhibition
BTLA	HVEM	· Inhibition of T cell activation, proliferation and cytotoxicity and cytokine production	· Inhibition of NK cell cytotoxicity
2B4	CD48	· Promotion of T cell activity or inhibition of T cell proliferation and function in a context-dependent manner	· Promotion of NK cell activity, cytotoxicity and cytokine production
OX40	OX40L	· Promotion of T cell proliferation, differentiation, IL-2 production, survival and the generation of memory T cells · Inhibition of Treg immunosuppressive function	· Poorly documented. · Promotion of NK cell cytotoxicity and IFN-γ production only seen in the context of simultaneous FcγR binding
4-1BB	4-1BBL	· Promotion of T cell proliferation, activation, IL-2 production, survival and the generation of memory T cells	· Promotion of NK cell proliferation · Conflicting results on NK cell cytolytic activities: no modulation or activation
ICOS	ICOSL	· Promotion of T cell proliferation, differentiation, cytokine production, function, survival and the generation of memory T cells	· Not documented for human NK cells · Promotion of NK cell maturation and effector functions in murine NK cells
GITR	GITRL	· Promotion of T cell proliferation, activation, differentiation, cytokine production · Inhibition of Treg immunosuppressive function	· Opposite role documented: promotion or inhibition of NK cell activation and effector functions

### 2.1 General expression and impact of immune checkpoints on T and NK cells’ function

#### 2.1.1 Programmed death protein 1

PD-1 (or CD279) is a receptor expressed by activated immune cells, mainly CD4^+^ and CD8^+^ T cells. It can interact with two different ligands: programmed death-ligand 1 (PD-L1) and PD-L2, themselves expressed mostly by antigen-presenting cells (APC), stromal cells or tumor cells (10, 11). PD-1/PD-L1 or PD-1/PD-L2 interaction is responsible for the downregulation of T cell activation, proliferation and function (12). In chronic viral infections or cancers, upregulation of PD-1 on T cells is considered as a feature of T cell exhaustion. The dampening of T cell response through PD-1 has been largely exploited by tumor cells who commonly express PD-Ls to escape T-cell-mediated anti-tumor immune response (10, 11, 13). Therefore, IC inhibitors targeting either PD-1 or PD-L1 have been widely used for the last decade in cancer immunotherapy (14). Yet, a recent study highlighted a dual function of PD-1 with PD-1-expressing tissue-resident memory CD8^+^ T cells having a protective role in patients with HCC (15). Moreover, PD-1 expression also prevents T cells from reaching the stage of terminally exhausted T cells and is necessary for the reactivation of exhausted T cells (12, 16, 17).

While it has been demonstrated that PD-1 play a role in the inhibition of NK cell cytotoxic activities and cytokine production (18–21), its degree of expression on NK cells remains controversial. PD-1 is only expressed at low detectable amount in the peripheral blood of healthy individuals, usually less than 5% of total NK cells (19–22), but the percentage of circulating PD-1^+^ NK cells seems to be increased in patients with different types of cancers as well as in some chronic viral infections (20). PD-1 mRNA as well as cytoplasmic full proteins were also found present within NK cells’ cytoplasm, suggesting that PD-1 can be rapidly externalized on NK cell surface (22). However, another recent study demonstrated that human and murine NK cells poorly express PD-1 which is not increased upon NK cell activation (23). Another group showed that PD-1 expression remains very low in resting human NK cells, but also revealed that PD-1 is more represented when looking at specific subsets of NK cells compared to the general NK cell population (24).

Metabolic fitness is crucial factor for immune cell functions. Usually, during activation, T cells undergo metabolic reprogramming allowing driving specific functional orientations. Interestingly, it has been demonstrated that ligation of PD-1 prevents engagement into glycolysis while promotes fatty acid oxidation and lipolysis, thus altering T-cell metabolic reprogramming (25). As glycolysis is required for T cells to differentiate into effectors, PD-1 blocks effector cell differentiation through metabolic perturbations. Similarly, NK cells expressing PD-1 exhibit compromised cellular metabolism, especially during chronic viral infections (26).

#### 2.1.2 Cytotoxic T lymphocyte antigen-4

Naive CD4^+^ and CD8^+^ T cells express TCRs that can specifically recognize antigens presented by APCs through their MHC II. TCR engagement with MHC II/antigen complex provides a first signal to initiate T cell activation. A second signal is mandatory for proper T cell activation, enabling the survival and proliferation of T cells. This co-stimulatory signal is mediated by the interaction between CD28 expressed by T cells and its ligands of the B7 family expressed by APCs: B7.1 (CD80) and B7.2 (CD86) (9). CTLA-4 (or CD152) is a receptor constitutively expressed on regulatory CD4^+^ FOXP3^+^ T cells (Treg) and rapidly induced on activating T cells which is closely related to CD28 in terms of structure but having opposite functions (27). CTLA-4 can bind B7 ligands with a better affinity than CD28, therefore limiting early T cell activation (28). CTLA-4 interaction with B7 ligands negatively regulates cell cycle progression and interleukin (IL)-2 production, blocking T cell activation, and can eventually lead to T cell anergy (9, 28).

The presence of CTLA-4 has been demonstrated in activated mouse NK cells, where it was responsible for the inhibition of interferon (IFN)-γ production by NK cells upon co-culture with mature dendritic cells (DCs) (29). However, whether CTLA-4 is expressed by human NK cells and its putative impact on NK cell function has been poorly documented. The initial study indicated that human NK cells do not express surface or intracellular CTLA-4, independently of their activation state (30). However, surface expression of CTLA-4 in *ex-vivo* activated human NK cells was detected more recently (31). Strikingly, the absence of CTLA-4 in human activated NK cells had a deleterious impact on their function with impaired degranulation activity and IFN-γ production (31). Using *in vitro* and in *ex vivo* approaches, Davis-Marcisak et al. demonstrated that both human NK cell lines and healthy human donor-derived NK cells expressed CTLA-4 on their cell surfaces and could bind anti-CTLA-4 antibodies (32). Another recent study observed low but present CTLA-4 expression in resting NK cells, with the highest expression of CTLA-4 found in the CD56^dim^ CD16^−^ NK cell subset (24). In addition, CTLA-4^+^ NK cells had reduced production of inflammatory cytokines IFN-γ and tumour necrosis factor (TNF) -α and reduced cytotoxicity (24). Importantly, CTLA-4^+^ NK cell population is enriched in liver compared to peripheral blood (17).

#### 2.1.3 T cell Immunoglobulin domain and mucin domain 3

TIM-3 (or CD366) is another receptor found on the surface of several immune cells including T cells and NK cells. Various ligands have been identified for TIM-3 such as phosphatidylserine (PS), high mobility group protein B1 (HMGB1), carcinoembryonic antigen-related cell adhesion molecule 1 (CEACAM1), and galectin-9 (Gal-9) (33). PS is also binding to other TIM proteins with better affinity than TIM-3, and its role in the regulation of T cell function has not been yet clarified (33). A recent study showed that TIM-3 bound to PS acts as a co-stimulatory receptor for TCR signaling in Jurkat cells (34). HMGB1 binds to TIM-3^+^ DCs and negatively regulates innate immune responses (35) but no evidence towards the modulation of T cell response by the HMGB1/TIM-3 axis has been reported. By contrast, TIM-3 interaction with Gal-9 and CEACAM1 has been shown to inhibit T cell immune responses and induce T cell apoptosis (36, 37). TIM-3 is known to be preferentially expressed on CD8^+^ T cells and the upregulation of TIM-3 has been linked with T cell exhaustion and bad prognosis in many types of cancer (38–41).

Unlike most non-classical NK cell inhibitory receptors, TIM-3 is expressed by resting NK cells and is further upregulated upon NK cell activation (42). TIM-3 was first described as a marker for mature NK cells playing a role in the promotion of IFN-γ production by NK cells (42, 43). However, TIM-3 cross-linking on NK cells was also associated with the impairment of NK cell cytotoxicity (43). Supporting these observations, TIM-3 blockade led to the improvement of NK cell effector functions in mouse models of HCC (44). So et al. showed that different stimuli could induce TIM-3 expression on NK cells and that the function of TIM-3 on NK cells might be context-dependent (45). Overexpression of TIM-3 on NK cells could also explain the inhibitory function attributed to this receptor in certain situations like chronic infections or cancer (46, 47).

#### 2.1.4 Lymphocyte activation gene-3

LAG-3 (or CD223) shares structural homologies with CD4 and interacts mainly with peptide bound-MHC II (48, 49). It can bind to four other ligands: fibrinogen-like protein 1 (FGL1), galectin-3 (Gal-3), α-synuclein fibrils (α-syn) and liver and lymph node sinusoidal endothelial cell C-type lectin (LSECtin). LAG-3 negatively regulates T cell activation, proliferation as well as cytokine secretion upon ligand binding (48, 49). Just as for PD-1, CTLA-4 and TIM-3, LAG-3 is known as a marker of T cell exhaustion and is found overexpressed in different types of cancers or chronic infections (50). The function of LAG-3 in NK cells has been less documented. The role of LAG-3 in murine NK cells was originally described using knock-out mice and appeared as a promoter of NK cell cytotoxicity against certain tumor cell lines (51). However, conflicting results were obtained with human NK cells where LAG-3 did not influence NK cell cytotoxicity (52). More recently, LAG-3 surface expression was demonstrated in human NK cells after exposure to IFN-α. LAG-3 blockade also revealed that LAG-3 is a negative regulator of cytokine production by mature NK cells, but does not affect NK cell cytotoxicity (53). This was supported by the study of Esen et al. in which LAG-3^+^ NK cells also displayed reduced production of inflammatory cytokines (24). On the other hand, they could observe reduced cytotoxic activities for LAG-3^+^ NK cells. Therefore, the role of LAG-3 in NK cell cytotoxicity still needs to be confirmed.

#### 2.1.5 T cell immunoreceptor with immunoglobulin and ITIM domain

TIGIT is a co-inhibitory receptor expressed by T cells and NK cells. TIGIT can bind to two different ligands expressed by tumor cells and APCs: CD155 (PVR), and CD112 (PVR2) with lower affinity (50, 54). In T cells, TIGIT interaction with ligands was shown to reduce T cell proliferation, activation, and function with impaired TCR signaling (50, 55). Furthermore, upregulation of TIGIT is observed in tumor infiltrating T cells, and TIGIT blockade improved T cell responses (50, 56, 57). TIGIT is significantly expressed by resting NK cells (24). DNAM-1 (or CD226) present in NK cells bind the same ligands as TIGIT, but with lower affinity. DNAM-1 acts as a co-stimulatory receptor to enhance NK cell cytotoxicity (58). However, TIGIT signaling provides opposite effects by reducing NK cell proliferation and cytotoxic capacities (57, 58). Surprisingly, a recent study demonstrated that cytotoxic activities in TIGIT^+^ NK cells remained unaffected (24). Nevertheless, upregulation of TIGIT in NK cells was associated with tumor progression in several studies and blocking TIGIT showed beneficial effects with the enhancement of NK cell effector functions (57, 59).

#### 2.1.6 B and T lymphocyte attenuator

BTLA belongs to the CD28 superfamily, and is expressed by T and B cells but also by NK cells and DCs. BTLA binds to herpes virus entry mediator (HVEM), a member of the tumor necrosis factor receptor (TNFR) superfamily, expressed by most haematopoietic, endothelial and epithelial cells (60, 61). Depending on the signaling pathway triggered, BTLA-HVEM interaction mainly exerts negative effects on proliferation, and drives inhibitory pathways and T-cell dysfunction, but it can also positively regulate T cells. BTLA is expressed on both CD4^+^ and CD8^+^ T cells and the expression levels are related to T cell stage of differentiation and activation processes, with the expression transiently increased upon activation, but decreased in activated T cells (61). In NK cells BTLA induces immunosuppression (62), mainly by inhibiting NK cell-mediated cytotoxicity (63).

#### 2.1.7 2B4

2B4, or CD244, belongs to signaling lymphocyte activation molecule (SLAM) family (64). 2B4 is expressed on NK and T cells and the engagement of 2B4 has been variably shown to activate or inhibit cell functions based on context and on the availability of downstream regulating proteins (65). Interestingly, 2B4 was discovered as a receptor implicated in anti-viral immunity. This was assumed from the observation that mutations of the SLAM-associated protein impaired 2B4-dependent stimulation of T and NK cell anti-viral functions (64). 2B4 seems to be important for stimulating NK cells and lowering the surface expression after SAP ligand-induced down-modulation results in reduced 2B4-mediated NK cell activation, cytotoxicity and overall cytokine production (66).

#### 2.1.8 OX40

OX40 (or CD134) is not expressed on naive T cells but is induced within 24-48 hours after TCR stimulation (67). Similarly, OX40-ligand (OX40L) is not constitutively expressed by APCs but induced upon activation. OX40 signaling enhances IL-2 production as well as expression of the IL-2 receptor α chain (CD25) by T cells and promotes T cell proliferation, differentiation, survival and the generation of memory T cells (67, 68). OX40 is known to be preferentially expressed on activated CD4^+^ T cells compared to CD8^+^ T cells. Importantly, OX40 is constitutively expressed in mouse FOXP3 Treg cells and rapidly induced in human Treg cells, where it acts as a negative regulator of Treg immunosuppressive function (68).

Interestingly, NK cells have been shown to express both OX40 (69–71) and OX40L (72, 73) but the role of OX40 in NK cells remains elusive. Turaj et al. demonstrated that the induction of OX40 on activated NK cells is dependent on the presence of activated T cells or monocytes, presumably due to the cytokines they secrete (70). In addition, they showed that OX40 engagement with its ligand alone was not sufficient to stimulate NK cell effector function. However, OX40 enhanced NK cell cytotoxicity and IFN-γ production in the context of simultaneous FcγR binding (70).

#### 2.1.9 4-1BB

4-1BB (or CD137) is a co-stimulatory molecule expressed by several immune cells upon their activation, including T cells and NK cells (74, 75). 4-1BB can bind to only one confirmed ligand named 4-1BB ligand (4-1BBL) which is present on the surface of activated APCs (74). This interaction provides stimulatory signals for T cells that promote IL-2 production, T cell proliferation, activation, survival and the generation of memory cells (74, 76, 77). The stimulatory properties of 4-1BB were broadly exploited in immunotherapy to potentiate the action of chimeric antigen receptor (CAR) T cells, which are genetically engineered T cells recognizing antigens without MHC restriction (78).

The effect of 4-1BB stimulation in NK cells is not fully understood. 4-1BB seems to play a role in NK cell regulation, as 4-1BB signaling led to NK cell reduction in several studies (79, 80). In fact, 4-1BB may be a receptor with both inhibitory and activating properties for NK cells (81). On one side, the 4-1BB signaling pathway induced impaired NK cell function in leukemias (81). On the other hand, 4-1BB could act as an activating receptor for NK cells as it was shown to promote NK cell proliferation (81–83) as well as cytolytic functions of NK cells (81). Whether 4-1BB is a stimulatory molecule for NK cell cytotoxicity remains controversial, as other recent studies demonstrated that NK cell cytotoxicity was not affected by 4-1BB signaling (82, 84). Therefore, the role of 4-1BB in NK cells needs to be clarified.

#### 2.1.10 Inducible T-cell co-stimulator

ICOS (or CD278) is a CD28 homolog rapidly induced on T cells after their activation. ICOS binds to its unique ligand belonging to the B7 family ICOS-ligand (ICOSL), also named B7H2 or CD275, which is constitutively expressed by APCs and can be induced in many other cell types such as cancer cells (85). After binding to ICOSL, ICOS provides stimulatory signals for T cell proliferation, function and survival and plays a role in the development of memory T cells (86–88). ICOS co-stimulation does not enhance IL-2 expression but rather both anti-inflammatory cytokines (IL-4, IL-5, IL-10 and IL-13) and pro-inflammatory cytokines (IL-6, IL-17, TNF-α and IFN-γ) expression, underlying the crucial role of ICOS for the development and regulation of CD4^+^ T helper (Th) subsets Th1, Th2, Th17 and Treg responses (85, 86, 89, 90). ICOS is also essential for the function and maintenance of follicular helper T cellss, another CD4^+^ T cell subset that promotes the development of high-affinity memory B cells in germinal centers (86, 91). Anti-ICOS agonists were already tested in mice studies and in clinical trials for cancer immunotherapy, and show promising results when used in combination with other checkpoint inhibitors like anti-PD-1 and anti-CTLA-4 antibodies (85, 92, 93).

Even though the role of ICOS has been widely described for T cells, few studies investigated its impact in NK cells and its function was only assessed in mice studies. ICOS expression was still demonstrated in NK cells from healthy human peripheral blood mononuclear cells (PBMC) where it was further upregulated in response to IL-2 + IL-12 stimulation (94). ICOS is also upregulated upon cytokine stimulation in murine NK cells and potentiates the cytotoxicity and IFN-γ production of activated NK cells (95). In line with this finding, ICOS-knockout mice had reduced amount of NK cells characterized by impaired cytotoxicity and IFN-γ production, together with a defective maturation of ICOS-deficient NK cells in the spleen and the bone marrow (96). These studies highlight the important role of ICOS in NK cell development and effector function, but the exact role of ICOS on human NK cells remains to be determined.

#### 2.1.11 Glucocorticoid-Induced tumor necrosis factor-related protein

GITR is constitutively expressed by Treg and presented at low levels on resting CD4^+^ and CD8^+^ T cells, where its expression is rapidly induced upon T cell activation (97). The association of GITR with its ligand, GITR ligand (GITRL), expressed by endothelial cells and APCs, provides opposite effects for Treg and other T cells. Indeed, GITR-GITRL interaction potentiates effector T cell response by suppressing Treg inhibitory activities and enhancing T cell activation, proliferation and cytokine production (97). In addition, GITR is crucial for the differentiation of several T cell subsets comprising Th9, Th17 and follicular helper T cells (97).

NK cells constitutively express GITR at low level that is further upregulated upon stimulation with cytokines or toll-like receptor-ligand (81). Whether GITR-GITRL interaction leads to stimulatory or inhibitory effects for NK cell function is still unclear. One study demonstrated a positive outcome after GITR engagement on NK cells with enhanced activation (98), cytotoxicity and IFN-γ production. Other studies indicated that GITR negatively regulates NK cell activation, proliferation, cytotoxicity and cytokine production (81, 99).

### 2.2 Role of immune checkpoints during anti-viral responses

The function of T cells is tightly regulated by both positive and negative signals to ensure the successful elimination of pathogens (viral clearance) while limiting immune-mediated pathology. In addition to tissue microenvironment, regulatory T cells or inhibitory cytokines, ICs are crucial in the regulation of the balance between immune-mediated viral clearance and maintenance of integrity to prevent immune-driven pathology. During acute viral infections, expression of inhibitory ICs is upregulated to limit the effector functions following T-cell activation, and subsequently downregulated upon viral clearance. Indeed, PD-1, CTLA-4, TIM-3, 2B4, LAG-3 and TIGIT are often co-expressed to limit the extent and duration of activated T cells’ action. However, in patients with chronic infections, these ICs are continuously expressed on virus-specific T cells, which causes dysfunction and exhaustion (100, 101). Immune suppression caused by IC upregulation enhances viral persistence and favours chronic infections. Many pathogens, including HBV, promote inhibitory interactions through ICs to escape immune control (102). Next, we will describe in detail the perturbations of the IC landscape in the context of HBV.

## 3 Dynamic and disturbances of immune checkpoints in T cells and NK cells during HBV infection

As mentioned previously, HBV infection is self-resolving in 95% of infected adults. Only a small proportion of patients progress to a chronic stage. Robust and highly functional HBV-specific T cells and NK cells are required for viral resolution. The frequency of anti-viral effectors then declines, and the residual T cell response remains for decades and insure long-term protection against persistent cccDNA in hepatocytes (103). On the other hand, in chronic infection, virus-specific T cells and NK cells become dysfunctional. The exact mechanism driving control of HBV infection or elicitation of chronicity remained undefined. Yet, ICs may be at the centre of T and NK-cell exhaustion.

IC molecules in the context of HBV are described mainly on the circulation level while only limited data exist about their expression on intrahepatic lymphocytes in HBV patients. The liver is an organ with a particular tolerogenic immune microenvironment. Indeed, it filters everyday large amounts of blood through the portal vein coming from the digestive system that is enriched in microbial products and food-derived antigens. The intrahepatic compartment is thus equipped to prevent excessive activation of the immune system and limit tissue injury. The cell composition, immunosuppressive soluble molecules and the abundance of inhibitory IC ligands are participating in maintaining liver homeostasis but can also be exploited by pathogens such as HBV to establish persistent infections (104, 105). It is thus of particular importance to better describe the immune status of liver-infiltrating lymphocytes at the main site of the HBV infection and determine whether it reflects what is observed in their peripheral counterparts.

Here we summarize the current knowledge about IC molecules on T and NK cells in the circulation and on the intrahepatic level, together with the expression of their ligands in the liver microenvironment, in HBV patients and contributions from animal models of HBV infection. Moreover, as ICs do not only exist as membrane-bound proteins but can also be found in soluble forms as a result of membrane shedding or alternative splicing, we included the existing information related to soluble IC (sIC) levels in HBV patients.

### 3.1 Immune checkpoint molecules’ modulation in the circulation during acute and chronic HBV infection

#### 3.1.1 Immune checkpoints on circulating T cells in HBV

**Figure 2:**
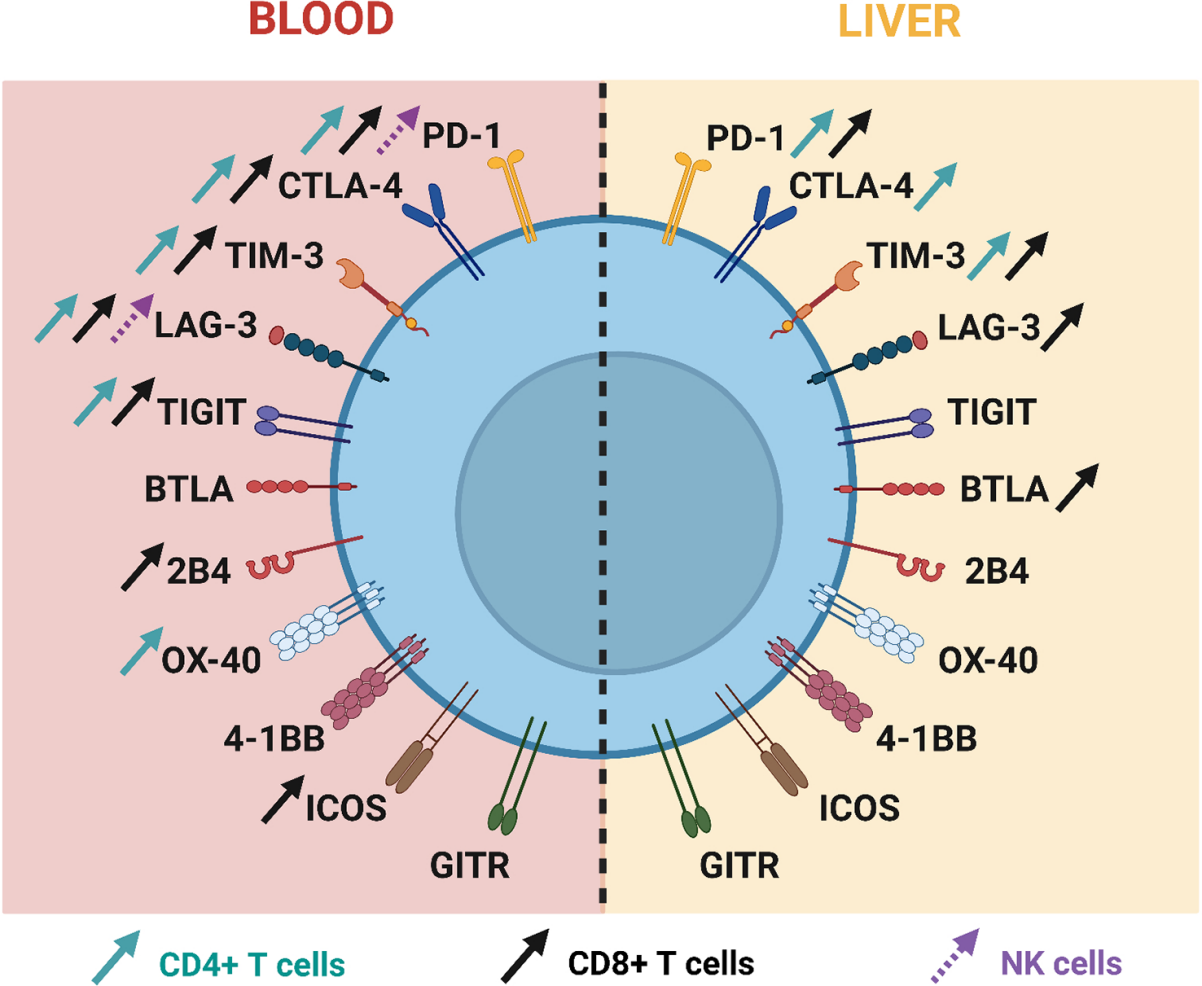
Current knowledge about immune checkpoint expression on T cells and NK cells during chronic HBV infection.

Following acute HBV infection, inhibitory ICs are upregulated on T cells and ICs expression is then downregulated on HBV-specific T cells following disease resolution, whereas their expression remains continuously high in patients who progress into the chronic stage (100, 102, 106), **Figure 2**. Such differences underline their key role in driving T-cell exhaustion in chronic HBV patients. The role of many ICs, such as PD-1, CTLA-4, TIM-3 but also TIGIT and BTLA, has been unveiled in this context (107).

PD-1 is one of the most studied inhibitory IC in HBV field. After initial T cell priming, PD-1 is upregulated on expanded HBV-specific CD8 T cells during the early phase of infection, and subsequently decreases following viral resolution (108). PD-1 expression on HBV-specific CD8 T cells attenuates HBV-specific effector function during the acute phase and correlates with the development of HBV-specific memory CD8 T cells during the resolution of infection (109). The initial upregulation of PD-1 allows to minimize excessive immune responses during acute inflammation and to prevent liver damage and intrahepatic inflammation. However, delayed/prolonged PD-1 expression on HBV-specific CD8 T cells was associated with acute liver failure (108). Many studies demonstrated that circulating total and HBV-specific CD4^+^ and CD8^+^ T cells overexpress PD-1 in patients with CHB (110–120). Notably, a genome-wide expression profiling of HBV-specific CD8 T cells from chronic HBV patients revealed multiple deregulated pathways, including mitochondrial dysfunction that was enriched in PD-1^hi^ CD8 T cells (121). Importantly, recent study demonstrated that PD-1^hi^ CD8^+^CXCR5^+^ follicular T cells represent effector rather than exhaustive phenotype in CHB (122) which highlights a dual function of PD-1. Additionally, in CHB patients, PD-1^hi^ expression was observed on circulating follicular helper T cells which was associated with the immune status of CHB (120, 123, 124).

CTLA-4 influences the vigour of T-cell response and the outcome of HBV infection as reviewed previously (125). Moreover, CTLA-4 gene polymorphism was associated with clinical recovery upon HBV infection (126). Indeed, specific single nucleotide polymorphisms have been associated with viral clearance, others with viral persistence. In patients with CHB, CTLA-4 is overexpressed at the basal state and following different stimuli in global circulating CD8^+^ T cells compared to healthy controls (127–129). Specifically, the frequency of CTLA-4^+^ HBV-specific CD8^+^ T cells is increased, and these cells express higher levels of CTLA-4 on their surface upon viral antigen stimulation (129). A similar trend was observed in another study where both the frequency and mean fluorescence intensity of CTLA-4 were upregulated on HBV-specific CD8^+^ T cells in hepatitis B e-antigen (HBeAg)^+^ patients compared to HBeAg^-^ patients (130). In addition, several reports noticed that circulating CTLA-4^+^ CD4^+^ T cells were also increased in CHB patients compared to healthy individuals (116, 128, 129) (127). Indeed, CHB patients are characterised by a higher proportion of circulating Treg compared to control patients and resolved HBV patients (131). This immune cell population constitutively expresses CTLA-4, which actively contributes to the elevated frequencies of CTLA-4^+^ CD4^+^ T cells in HBV patients. HBV-related upregulation of the CTLA-4 expression on Treg cells promotes their suppressive function, mainly *via* IL-10/TGF-β signalling (125, 132)

TIM-3 is another classical T cell inhibitory IC that was widely studied in HBV. In acute HBV infection, TIM-3 is upregulated on total CD4 and CD8 T cells compared to healthy donors and further decreases during convalescent phase (133). In the peripheral blood of CHB patients it has been shown that TIM-3 is highly expressed by both CD4^+^ and CD8^+^ T cells and linked with disease severity (114, 120, 133–136). Increased TIM-3^+^ T cells were associated with decreased plasma IFN-γ level in CHB patients and negatively correlated with T-bet mRNA expression (133), which is supported by *ex vivo* experiments highlighting a defective production of IFN-γ upon TIM-3^+^ CD8^+^ T cell stimulation (134). Moreover, the overexpression of TIM-3 is involved in disease progression of CHB through skewing of Th1/Tc1 responses, contributing to persistency of HBV infection (133).

Compared to the well-known receptors PD-1, CTLA-4 and TIM-3, the expression and role of LAG-3 in CHB has been much less investigated. Yet, LAG-3 expression on circulating CD4^+^ and CD8^+^ T cells from CHB patients was found to be significantly higher compared to healthy controls, and the frequencies of LAG-3^+^ T cells positively correlated with alanine aminotransferase (ALT) levels (117, 137). Moreover, the studies revealed that LAG-3^+^ T cells have reduced ability to secrete pro-inflammatory cytokines (117, 137). However, there was no difference between LAG-3 mRNA expression from peripheral blood CD8^+^ T cells of HBV-related HCC patients compared to the one of healthy controls (138).

The contribution of TIGIT in HBV has recently gained significant attention. In chronic HBV infection, TIGIT expression is upregulated by both circulating CD4^+^ and CD8^+^ T cells, which positively correlates with ALT and HBV viral load (139). These TIGIT expressing T cells have impaired ability to secrete pro-inflammatory cytokines upon antigen stimulation compared to their TIGIT negative counterparts (139). Similarly, it has been observed that the frequencies of peripheral TIGIT^+^ CD4^+^ and CD8^+^ T cells co-expressing PD-1 are increased in HBV-related HCC patients, and correlate with advanced stage of the disease and poor prognosis (140). In hepatitis B surface antigen (HBsAg) transgenic mice, TIGIT blockade leads to emergence of HBsAg-specific T cells, chronic hepatitis and progression to HCC, suggesting a role for TIGIT in maintaining immune tolerance to HBsAg in this model (141).

While a recent study showed increased levels of both BTLA and HVEM on circulating CD4^+^ and CD8^+^ T cells of CHB patients compared with healthy controls (142), no difference was observed in earlier studies (143, 144). As mentioned previously, the expression levels of BTLA are related to the lymphocyte stage of differentiation. In HBV infection, BTLA is highly expressed in a subset of virus-specific T-cells, which can potentially have an inhibitory effect on T-cell proliferation and cytokine secretion (145, 146). In addition, BTLA genetic variants seem to influence the susceptibility to severe chronic hepatitis B (147).

The circulating frequency of 2B4 HBV-specific CD8^+^ T cells has been found to be elevated in chronic HBV patients compared to patients who resolved the infection and the blockade of 2B4 restored the function of HBV-specific T cells *in vitro* (148).

OX40/OX40L is another IC pathway that seems to contribute to effective HBV clearance. The study from Publicover et al. demonstrated that peripheral CD4^+^ T cells from patients with acute HBV were enriched compared to CHB patients and displayed increased expression of OX40 compared to both CHB patients and healthy individuals (149). In patients with chronic HBV infection, circulating HBV-specific CD4^+^ T cells expressed higher levels of OX40 compared to the global CD4+ T cell population, and the large majority of HBV-specific CD4 T co-expressed OX40 and PD-1 (119).

Limited information exists about co-stimulatory molecule 4-1BB in chronic HBV infection. Even though no statistical difference was underscored, 4-1BB mRNA expression levels in CD4^+^ and CD8+ T cells of CHB patients appeared higher than the ones in healthy donors (150). 4-1BBL was also upregulated in circulating monocytes from CHB patients and was closely associated with the development of liver cirrhosis, which raises the possibility that T cells might be excessively stimulated *via* 4-1BB in chronic HBV infection (151).

Levels of ICOS have also been reported to be increased in peripheral CD8^+^ T cells of CHB patients compared to healthy donors (116). ICOS levels negatively correlated with HBV DNA, suggesting that higher frequencies of ICOS^+^ CD8^+^ T cells favour viral control during CHB (116). Similarly, Hu et al. observed that ICOS^+^CXCR5^+^CD4^+^ T cells were negatively correlated with HBV DNA loads (123).

Little is known about GITR expression in patients with CHB. Interestingly, in CHB patients who spontaneously underwent HBsAg seroconversion, the proportion of total GITR^+^ CD4^+^ T cells and of GITR^+^ CD8^+^ T cells were decreased compared to HBsAg positive patients (152). Therefore, the contribution of GITR in anti-HBV immune response still needs to be clarified.

Co-expression of multiple ICs on T cells has been highlighted in chronic HBV patients. PD-1 is often co-expressed with other inhibitory receptors, which may play non-redundant roles in order to avoid excessive activation of the immune system. Indeed, in CHB patients, higher frequencies of HBV-specific CD8^+^ T cells positive for PD-1, LAG-3 and TIM-3 were associated with a lack of functional response (153). Patients in the active phase also displayed increased percentages of PD-1^+^ TIM-3^+^ TIGIT^+^ CD8^+^ T cells (152). Besides, it has been described that PD-1 and 2B4 are highly co-expressed on HBV-specific CD8 T cells (148). Contrary to CD8^+^ T cells, CD4^+^ T cells from chronic HBV patients harboured high levels of PD-1 but without upregulation of other inhibitory ICs (CTLA-4, TIM-3, 2B4) (115). However, levels of PD-1, CTLA-4 and TIM-3 are enhanced on Treg during HBV infection and contribute to their regulatory functions (125, 154). All these elevated levels of ICs are associated with loss of cytokine secretion by T cells which contribute to T-cell exhaustion and progression into the chronicity.

#### 3.1.2 Immune checkpoints on circulating NK cells in HBV

PD-1 expression on NK cells in the context of HBV has been much less investigated. A study focused on NK cells purified from PBMC revealed significantly higher levels of PD-1 compared to healthy individuals (155). Furthermore, NK cells from CHB patients exhibit a regulatory phenotype by producing more IL-10 upon stimulation, which in turn inhibits CD4^+^ and CD8^+^ T cell activation (155). Marotel et al. observed only slightly upregulated PD-1 expression in peripheral NK cells from HBV patients compared to healthy individuals and linked the NK dysfunction in CHB to deregulated calcium signalling (156). Functional defects have also been reported in PD-1^+^ TIGIT^+^ NK cells among CHB patients (157).

The contribution of CTLA-4 on NK cells remains to be determined, and in this regard, no study investigated the role and expression on NK cells in the context of HBV infection so far.

Concerning TIM-3, there is a trend for circulating NK cells from CHB patients to display increased TIM-3 expression compared to levels in control group which correlated with serum ALT levels (158). This was recently challenged by a study from Marotel et al. showing that surface expression of TIM-3 on peripheral NK cells is reduced in CHB patients compared to healthy controls (156). It is still needed to shed light on the expression and impact of TIM-3 in NK cells during CHB, as TIM-3 signalling may modulate NK cell function in the disease context. Interestingly, Liu et al. explored the expression of TIM-3 ligand Gal-9 by NK cells, and found that Gal-9 is upregulated in NK cells in CHB patients compared to healthy control, which was associated with functional impairment of NK cells characterized by co-expression of inhibitory ICs PD-1 and TIGIT and impaired cytokine and granzyme B production (157). Increased Gal-9 was also shown at the mRNA level in circulating NK cells and in the serum of CHB patients compared to healthy controls (157). Importantly, Gal-9 expression was significantly reduced after antiviral therapy. Co-culture experiments of CD8^+^ T cells with Gal-9^+^ or Gal-9^-^ NK cells demonstrated that NK cells could impair CD8^+^ T cell function in a TIM-3/Gal-9 dependent manner highlighting a novel role for NK cells in regulating CD8^+^ T cell response (157). Finally, an increased frequency of NK cells co-expressing TIM-3 and TIGIT is seen in HBV-related HCC and induces NK cell dysfunction (159).

In circulating NK cells, LAG-3 mRNA levels were found to be highly upregulated in CHB patients and surface expression was also greater compared to healthy controls (156). Nevertheless, the role of LAG-3 in NK cells is not well understood and data are still scarce, especially in the context of CHB. In CHB and HBV-HCC patients respectively, the co-expression of TIGIT and PD-1 or TIGIT and TIM-3 on NK cells is also elevated, and these specific subsets of NK cells display impaired function (157, 159). Elevated TIGIT expression on the surface of NK cells was also highlighted in CHB patients by Marotel et al. (156). Interestingly, another recent study investigated the role of TIGIT on circulating NK cells in CHB patients based on the disease status and demonstrated that TIGIT was overexpressed in NK cells of patients in the immune control phase, but that the proportion of TIGIT^+^ NK cells was much lower in NK cells of patients in the immune active phase compared to healthy individuals (160). Moreover, TIGIT^-^ NK cells had improved IFN-γ secretion, degranulation and cytotoxicity capacities compared to TIGIT^+^ NK cells, providing the hypothesis that TIGIT might prevent NK cells from causing liver injury.

Concerning 2B4, its expression on circulating NK cells is decreased during the early phase of HBV infection, resulting in impaired function of these cells (161).

#### 3.1.3 Soluble ICs in serum of HBV patients

Many cell surface ICs undergo alternative splicing to produce soluble isoforms, soluble ICs (sICs), which can be tracked in the serum of patients. One presumable mode of action of sICs is to regulate the function of their membrane isoforms counterparts through competitive binding.

Soluble PD-1 (sPD-1) and soluble PD-L1 (sPD-L1) levels are markedly increased in CHB patients compared to healthy donors (162, 163) and are associated with viral replication markers and liver inflammation (162, 164). In another study, only HBeAg^+^ patients had higher sPD-1 compared to healthy controls (165). Interestingly, sPD-1 is particularly increased in patients in the clearance or immune active phase, suggesting that sPD-1 may participate in the promotion of antiviral responses (162, 164, 165). sPD-1can interfere with the PD-1/PD-L1 or PD-1/PD-L2 pathway and enhance antitumor immunity (166, 167). However, elevated levels of sPD-1 can also reflect the state of the disease and the immune response against the viral infection. In this regard, high sPD-1 levels were associated with increased risk of developing HCC (164, 168, 169). Sex differences were noticed in terms of blood sPD-1 levels in HBV-related HCC patients where higher levels were evaluated in males compared to females (168).

The soluble form of CTLA-4 (sCTLA-4) can be also generated by alternative splicing and found free in circulation. Unlike sPD-1, sCTLA-4 does not improve T cell response by reversing T cell inhibition but rather contributes to the impairment of T cell response by binding to B7 ligands on APCs and preventing the co-stimulatory interaction between CD28 in T cells and B7 ligands (166, 167). Using different detection methods, two studies had divergent findings regarding sCTLA-4 concentrations in CHB patients compared to healthy donors. Either no difference was observed between the two groups using multiplex Luminex immunoassay (170), or sCTLA-4 concentration was found to be higher in CHB patients than healthy controls when measured by ELISA, and these levels correlated with ALT (171). Therefore, the involvement of sCTLA-4 and its levels in HBV patients need to be further defined.

As Gal-9 circulates in the serum in a soluble form, soluble TIM-3 (sTIM-3) can also be found in the circulation, but its role in the disease is not yet clarified. In CHB patients, sTIM-3 levels are increased in the serum compared to healthy controls, and are associated with a higher viral burden and liver inflammation (170, 172, 173). Moreover, when looking at different stages of the disease from inactive hepatitis to liver cirrhosis and cancer, sTIM-3 levels rise gradually suggesting a close association with the disease progression (172, 173). However, antiviral treatment did not modulate sTIM-3 levels in treated patients which were rather more elevated in the NUC-treated group compared to the non-treated one (170). Nevertheless, Gal-9 soluble form is also increased in the serum of CHB patients (135).

The role of soluble LAG-3 (sLAG-3) in hepatitis B infection is poorly investigated. One report revealed that the levels of sLAG-3 were not different in CHB patients compared to healthy controls (170).

Soluble levels of OX-40 and OX-40L were reported to be higher in CHB patients compared to healthy donors and the levels correlated with the virologic status of CHB patients (174).

Soluble 4-1BB (s4-1BB) levels were increased in the plasma of untreated CHB patients compared to healthy individuals (150). However, no difference in plasma levels of s4-1BB was seen between untreated and NUC-treated CHB patients. These higher concentrations of s4-1BB in CHB patients were positively correlated with viral replication markers but not with inflammation markers (150).

There are conflicting results regarding soluble ICOS (sICOS) serum concentrations in CHB. One study revealed the decreased levels of sICOS that were observed in CHB patients compared to control individuals, and the levels were not impacted by NUC treatment (170). Another study reported that sICOS levels are increased in both HBeAg^+^ and HBeAg^-^ CHB patients compared to healthy controls (165). A putative role of sICOS is to impair T cell activation by competing with ICOSL expressed by APCs, but it remains to be clarified.

The clinical significance of soluble GITR (sGITR) is not well defined in chronic HBV infection, but its concentration in the blood was found to decrease in CHB patients compared to healthy controls, as well as soluble GITRL levels, without any correlation with viral or inflammation markers (170).

### 3.2 Immune checkpoint modulation within the liver microenvironment during HBV infection

#### 3.2.1 Immune checkpoints on intra-hepatic T cells during HBV infection

Globally, intra-hepatic T cells display a more exhausted phenotype compared to circulating cells, with a strong expression of inhibitory receptors, associated with functional defects (reduced proliferative and cytotoxic functions). This state is maintained by continuous exposure to HBV antigens. Significant upregulation of PD-1 was observed in intrahepatic lymphocytes including CD4^+^ and CD8^+^ T cells from patients with CHB and HBV-related HCC (111–113, 175–178). The frequencies of PD-1^+^ HBV-specific CD8^+^ T cells in the liver compartment are known to be higher than in the peripheral blood, regardless of T cell differentiation stage (111). Of note, PD-L1 and PD-L2 are also overrepresented in inflamed liver tissue from CHB patients which can contribute locally to promote immune suppression through PD-1 engagement (112, 175–177) and the levels of PD-1, PD-L1 and PD-L2 in the liver were shown to correlate with the degree of inflammation (112, 176, 177). In a mouse model of HBV infection, it has been demonstrated that intrahepatic PD-1-expressing CD8^+^ and CD4^+^ T cells are increased in mice with HBV persistence, but PD-1 upregulation was resolved in mice which had cleared HBV (179).

Of note, an increased frequency of CD4^+^ CD25^+^ FOXP3^+^ Treg was also observed at the site of the liver in patients with a high viral load (180). These intrahepatic Treg displayed a different phenotype than their circulating counterparts, with notably higher expression of CTLA-4 (180). Besides dampening T cell activation, CTLA-4 upregulation in HBV patients also contributes to T cell dysfunction by skewing CD4^+^ T cell differentiation toward the Th2 and Treg phenotype, therefore favouring the secretion of anti-inflammatory cytokines (125). Moreover, transcriptomic analyses recently revealed that the livers of patients in the immune active phase and in HBeAg^-^ active hepatitis phase display upregulated genes involved in T-cell exhaustion, including CTLA-4 (181).

Liver tissues from CHB patients also displayed increased frequencies of TIM-3^+^ CD4^+^ and TIM-3^+^ CD8^+^ T cells compared to healthy controls (135, 158). In patients with HBV-related HCC, there was an increased proportion of TIM-3^+^ intrahepatic CD4 and CD8 lymphocytes in the tumoral region of the liver (178, 182). Interestingly, this was not observed in patients with alcohol-related HCC (182). Gal-9, ligand of TIM-3, was observed to be strongly expressed by the resident macrophages (Kupffer cells) in the liver of HBV-related HCC and CHB patients with active hepatitis (135, 182). These elements suggest a crucial role of the TIM-3/Gal-9 pathway in T cell dysfunction in patients with CHB.

LAG-3 mRNA expression was increased in tumor-infiltrating CD8^+^ T cells from HBV-related HCC patients compared to controls which was associated with lower capacity to produce IFN-γ (138). Whether LAG-3 expression is enriched or not in the liver of CHB patients remains to be determined.

In patients with HCC (including several ones with a background of HBV infection) the frequencies of GITR^+^ NK cells and CD8^+^ T cells were unchanged among tumor, non tumor liver tissue and blood while a small increase of the proportion of GITR expressing Tregs was seen in both tumor and non tumor compared to blood (183).

Chronic HBV infection is more likely to develop in patients infected perinatally or during childhood rather than in adults who spontaneously resolve the infection for the vast majority. Publicover and colleagues developed young and adult HBV transgenic mouse models in order to investigate on the contribution of immature and mature immune system in the clearance of the infection, and notably focused on the OX40/OX40L axis. They found that OX40L is much more expressed by hepatic APCs in adult mice compared to young mice (149). In particular, adult mice express more hepatic CD4^+^ T cells than young mice, and among them a greater frequency of OX40^+^ CD4^+^ T cells. Accordingly, OX40L mRNA expression is also expressed in an age-dependent manner in human liver when comparing infant liver and adult liver tissues (149).

Wang et al. reported the enrichment of BTLA-expressing HBV-specific CD8^+^ T cells in the liver of HBV patients, with an immune-regulatory role through IL10 secretion (146). In fact, intrahepatic T cells expressed higher levels of BTLA than their peripheral counterparts. In addition, significant fraction of intrahepatic T cells also co-expressed BTLA and PD-1 and show deep exhaustion of T cell responses (143).

In chronic HBV infection, intra-hepatic virus-specific CD8^+^ T-cells also expressed higher levels of 2B4 in comparison to the acute phase of infection or following resolution (148).

Besides, RNA sequencing of liver samples from CHB patients and healthy individuals revealed that several genes involved in immune activation including ICOS were upregulated in patients with active hepatitis but not in patients in the immune tolerant or immune control phase compared to healthy donors (181).

#### 3.2.2 Immune checkpoints on intra-hepatic NK cells during HBV infection

IC expression on intra-hepatic NK cells in the context of HBV has been much less investigated. Interestingly, Zhou et al. described that in mice, the intrahepatic NK cells control the antiviral activity of intrahepatic T cells *via* the PD-1/PD-L1 axis (184) but the specific information for HBV context is missing. Additionally, it has been reported that the cytotoxic activities of intrahepatic NK cells are sex dependent in HBV patients (185).

In HBV transgenic mice, where NK-cell mediated liver injury was induced by stimulation with concanavalin A, OX40L^+^ NK cells expanded in the liver as well as OX40^+^ Tregs upon liver injury triggering (186). NK cell-mediated hepatocytotoxicity was markedly reduced by Tregs through membrane-bound TGF-β and OX40/OX40L in a cell to cell contact dependent manner, which suggests a novel mode of action of Treg in the regulation of NK cell function in the liver (186). However, the role of the majority of ICs has never been uncovered in the context of intrahepatic NK cells in HBV infection and there is a clear need for future studies.

### 3.3 Link of immune checkpoint expression with clinical parameters and outcome

The frequency of PD-1-expressing total CD4/CD8 and HBV-specific CD8 T cells positively correlates with ALT levels in CHB patients (108, 114, 117). Moreover, in chronic HBV patients who spontaneously controlled the infection, i.e. patients who underwent HBsAg seroconversion without treatment, the frequency of global circulating PD-1^+^ CD4^+^ T cells was lower than in HBsAg^+^ patients, and the reduced expression of PD-1 was especially seen at the levels of Treg (152). This was confirmed by another study where PD-1 expression was decreased in Treg compared to other T cells in asymptomatic HBeAg^-^ chronic carriers (120). Moreover, higher levels of T cells expressing both PD-1 and TIGIT were associated with the progression of the disease in HBV-related HCC patients (140). PD-1 and mostly BTLA play an important role in the progression of CHB, as their expression levels are significantly upregulated during the progression of CHB from liver cirrhosis to HCC (187). CTLA-4 gene polymorphism determines recovery upon HBV infection (126). In addition, a positive correlation between TIM-3 expression on circulating T cells and markers of liver injury (ALT, aspartate aminotransferase (AST)) has been reported (133). Thus, the intensity of IC expression may be directly linked with the disease stage and severity of liver damage, further sustaining the role of ICs in the progression of the disease.

### 3.4 Impact of current anti-viral treatments on immune checkpoints expression on T and NK cells

Modulation of ICs expression during standard antiviral therapies could be associated with HBV elimination, highlighting their importance in immune-mediated viral clearance. In fact, as antiviral therapies for HBV suppress the viral replication, and T-cell exhaustion is driven by prolonged exposure to viral antigens, the decrease of viral antigens induced by standard antiviral therapies restored the functionality of virus-specific effectors cells. This was attested by several studies highlighting increased HBV-specific T-cell proliferation and cytotoxicity upon lamivudine treatment (188) or improved cytokine production following adefovir administration (189).

NUCs induce prolonged suppression of viral replication in chronic HBV patients as witnessed by a clear reduction of serum HBV DNA levels in NUC-treated patients. Interestingly, the levels of PD-1 on circulating T cells are normalized in patients who underwent NUC therapy (telbivudine, tenofovir or lamivudine) compared to untreated HBV patients, and this reduction is accompanied by improved ability of T cells to secrete IL-12 and IFN-γ (114, 127, 190–193). By contrast, a study including 28 HBV patients treated with telbivudine monotherapy did not highlight any significant decrease of PD-1 surface expression by circulating CD4^+^ T cells after 6 months of treatment (194). Nonetheless, PD-1 expression was also reduced in the liver biopsies of another cohort of patients 96 weeks after the start of the treatment compared to baseline (195). Importantly, NUC-treated patients had increased levels of circulating PD-1 compared to non-treated HBV patients (170). Nevertheless, serum concentrations of sPD-1 are reduced throughout the course of the treatment (163). A recent study revealed that the patients with detectable sPD-1 after NUC therapy were more likely to achieve HBsAg loss and had a reduced risk of clinical relapse compared to patients with undetectable sPD-1 following NUC discontinuation (196). Interestingly, T-cells from NUC-treated patients respond better to PD-1 blockade (115).

There are conflicting results regarding the effect of antiviral therapy on CTLA-4 expression on peripheral CD4^+^ and CD8^+^ T cells. It was previously shown that the frequencies of CTLA-4^+^ CD8^+^ T cells were not modulated after more than 1 year of NUC treatment (129). However, recent studies show a different trend, with sustainably reduced levels of CTLA-4 on CD8^+^ T cells (127, 193). By contrast, the proportion of CTLA-4^+^ CD4^+^ T cells seems to be enhanced with antiviral treatment (114, 193) or not modulated after 1 year of therapy (127). In liver biopsies from CHB patients, gene expression of CTLA-4 was also reduced in NUC-treated patients compared to untreated ones (195).

The effects of antiviral therapy on reducing TIM-3 levels on CD8^+^ T cells have been demonstrated in treated CHB patients compared to non-treated ones (114, 133, 157). However, these effects were less conclusive regarding TIM-3^+^ CD4^+^ T cells, where one study pointed out that there is reduced expression of TIM-3 on CD4^+^ T cells after 3 to 6 months of therapy (133), whereas no specific effect of NUC was observed for this parameter in two other reports (114, 193). Similarly, it was recently demonstrated that also the TIGIT gene expression is reduced in NUC-treated patients after 96 weeks of therapy compared to non-treated CHB patients (195). Contrary to NUC treatment, PEG-IFN therapy does not seem to modulate ICs on effector cells, as HBV-specific T cells still displayed an exhausted phenotype with unchanged PD-1 and CTLA-4 levels, irrespective of the treatment outcome (197).

## 4 Clinical exploitation of IC's for anti-HBV therapies

It is essential to better understand the immune status of CHB patients to consider appropriate immunotherapeutic approaches to control persistent HBV infection, alone or combined with antiviral agents. ICs play an essential role in the pathogenesis of chronic viral infections. Indeed, T-cell and NK-cell exhaustion mediated by ICs are important factors determining viral chronicity. It appears that the reshaping of ICs’ landscape on T and NK cells is a strategy used by HBV to escape immunity and establish chronicity, through the loss of functionality and exhaustion of these key anti-viral effectors. Targeting these pathways in chronically infected patients emerges as a promising strategy to overcome chronicity (101, 103, 107, 198). The goal of targeting ICs is to reactivate the host immune system by promoting immune cell function and getting over exhaustion, either by blocking inhibitory pathways or by engaging stimulatory molecules. Indeed, IC blockers will reinvigorate the function of pre-existing antiviral immunity. However, such immunotherapeutic approaches must be tightly monitored due to the high potential of immune activation, which can lead to liver damage and autoimmunity. Therefore, the ideal immunomodulatory intervention should promote an effective anti-viral response without exacerbating liver immunopathology. As discussed above, recent evidence highlights the role of inhibitory receptors in inducing NK cell dysfunction. This is why immunotherapies, which are often thought to benefit only from enhanced T cell response, can also promote NK cell function and favour viral control or anti-tumor immunity (199).

### 4.1 Pre-clinical evidence supporting the use of immune checkpoint blockade to reinvigorate T-cell and NK-cell mediated anti-HBV immunity

IC blockade emerged as a promising therapeutic option to restore T- and NK-cell function in the context of chronic HBV infection. Such strategies have been evaluated *ex vivo* from the blood of chronic HBV patients and *in vivo* in animal models of HBV infection (mice and woodchucks (Marmota monax) and its HBV-like woodchuck hepatitis virus) and reported benefits of targeting ICs alone or in combination in the context of HBV infection to increase HBV-specific CD8 T-cell function. The main findings about IC blockade in the context of chronic HBV infection are recapitulated in **Table 2**.

**Table 2:** Effects of immune checkpoint blockade on effector immune cells in chronic HBV infection and related diseases.

Disease	Targeted ICs	Target species	Model	Findings	References
HBV	PD-L1	Human	*In vitro*	· Enhanced proliferation and function of circulating and liver-infiltrating HBV-specific T cells	(110, 111)
HBV	PD-L1	Humans	*Ex vivo*	· HBV-specific CD8+ T cells response is enhanced only in patients with lower exhaustion levels (LAG-3- TIM-3- PD-1+) and not in patients with a higher frequency of functionally exhausted T cells (LAG-3+ TIM-3+ PD-1+)	(153)
HBV	PD-L1, PD-L2, CTLA-4, TIM-3	Humans	*Ex vivo*	· Partial restoration of cytokine secretion by CD4+ T cells and enhanced CD4+ T cell proliferation upon PD-L1/2 blockade · Blockade of CTLA-4 and TIM-3 alone fail to reactivate CD4+ T cell function	(115)
HBV	PD-L1, LAG-3	Humans	*Ex vivo*	· LAG-3 blockade improves IFN-γ secretion by CD8+ T cells · LAG-3 combined with PD-L1 blockade is more efficient to promote CD8+ T cell function compared to the use of anti-PD-L1 alone	(137)
HBV	PD-1, LAG-3	Humans	*Ex vivo*	· Combined blockade of PD-1 and LAG-3 improved the secretion of cytokines by circulating Th1 CD4+ T cells and reduced IL-10 secretion by CD4+ T cells as well as Treg expansion	(117)
HBV	PD-L1, PD-L2, TIM-3	Humans	*In vitro*	· Blocking TIM-3 improves HBV-specific CD8+ T cell responses · Dual blockade of TIM-3 and PD-L1/2 leads to synergistic effect by enhancing HBV-specific CD8+ T cells in some patients	(135)
HBV	PD-L1, OX40	Humans	*In vitro*	· Anti-OX40 or anti-PD-L1alone do not increase the percentage of functional peripheral HBV-specific CD4+ T cells upon stimulation with viral antigens · Combination of PD-L1 blockade and OX40 stimulation synergistically improve HBV-specific CD4+ T cell responses	(119)
HBV	PD-L1, CTLA-4	Humans	*Ex vivo*	· Combined blockade of PD-L1 and CTLA-4 boost effector T cell proliferation but only enhance IFN-γ production in HBeAg+ patients · No such effects are seen with anti PD-L1 or anti-CLTA-4 alone	(130)
HBV, LMCV	PD-L1, 4-1BB	Humans, mice	*Ex vivo*, *in vivo*	· Improvement of cytokine production by HBV-specific T cells in HBV-infected livers of patients · Enhanced proliferation, function and anti-viral efficacy of LMCV-specific CD8+ T cells · Better responses when monitoring the doses of anti-4-1BB towards lower ones to avoid overstimulation and cell death	(200, 201)
HBV	PD-L1, TIM-3, CTLA-4, LAG-3	Humans	*In vitro*	· PD-L1 blockade efficiently promotes circulating CD8+ T cell expansion unlike blockade of other inhibitory pathways · Combination of PD-L1 and TIM-3 blockade do not potentiate CD8+ T cell proliferation which is even reduced	(113)
HBV	CTLA-4	Humans	*In vitro*	· CTLA-4 blockade enhances peripheral and intrahepatic HBV-specific CD8+ T cell responses	(129)
HBV	TIM-3	Humans	*Ex vivo*	· Antiviral efficacy of peripheral virus-specific CD8+ T cells is only promoted in patients with robust pre-existing response to HBV peptide stimulation · TIM-3 blockade enhances cytotoxicity and IFN-γ production in NK cells	(134, 135, 158)
HBV-HCC	TIM-3	Humans	*Ex vivo*	· Enhanced proliferation and cytokine secretion of intrahepatic T cells	(182)
HBV	TIM-3, Gal-9	Humans	*In vitro*	· Effector functions of peripheral blood HBV-specific T cells are restored upon Gal-9 or TIM-3 blockade · Gal-9 blockade require NK cells in the culture to efficiently boost T cells, indicating that NK cells directly regulate T cells *via* the TIM-3/Gal-9 pathway	(157)
HBV	TIGIT	Humans	*In vitro*	· Partial restoration of cytokine secretion by HBV-specific CD8+ T cells	(139)
HBV	OX40	Mice	*In vivo*	· Improved anti-viral efficacy and T cell responses in young mice and adult chronic mice infected during youth, not seen with control mice treated with isotype antibody	(149)
HBV	4-1BB	Mice	*In vivo*	· Promotion of liver disease progression towards end stage HCC in HBV-transgenic mice, effects mainly mediated by CD8+ Tcells	(151)
LMCV	GITRL	Mice	*In vivo*	· Reversal of CD4+ and CD8+ T cell exhaustion by promoting early activation and enhanced effector function of T cells	(202)

PD-1 blockade has been investigated in mouse and WHV models of HBV infection. In mice hydrodynamically injected with the pAAV/HBV1.2 plasmid, anti-PD-1 led to HBV clearance and reversion of the exhausted phenotype of HBV-specific CD8 T cells by promoting IFNy secretion by HBV-specific intra-hepatic T cells (179). In the WHV model, anti-PD-L1/L2 antibodies partially restored T-cell function (203). The combination of antivirals, vaccination and PD-L1 blockade can lead to complete viral clearance (204–206). TIGIT blocking or deficiency in HBsAg transgenic mice leads to chronic hepatitis and fibrosis, along with the emergence of functional HBsAg-specific cytotoxic T lymphocytes (CTLs) (141), suggesting the breakdown of adaptive immunotolerance by TIGIT blockade or deficiency. TIGIT is critical in the maintenance of liver tolerance by keeping CTLs in homeostatic balance.

Besides, the blocking of PD-1 (111, 112, 115), CTLA-4 (129), TIM-3 (133, 135) or 2B4 (148) improved expansion and function of HBV-specific patient-derived CD8^+^ T cells *ex vivo*, favouring T-cell proliferation, cytokine secretion and cytotoxicity. Whereas PD-L1 blockade can also enhance CD4^+^ T-cell proliferation and functionality (115), the blockade of other ICs does not have any impact. Interestingly, the responsiveness of HBV-specific CD8^+^ T cells from chronic HBV patients to *ex vivo* PD-L1 blockade depends on the differentiation stage of T cells, the most responsive being effector memory T cells in intermediate differentiation stage (CD27^+^ CCR7^-^ CD45RA^-^) (113). In addition, the *ex vivo* blockade of the BTLA pathway enhanced both intrahepatic and PBMC T cell proliferation and cytokine secretion, and exhibited an additive effect upon blockage of PD-1 (143).

The triggering of activating ICs using agonists is also under investigation. Indeed, using GITRL transgenic mice, Pascutti et al. showed that increased co-stimulation through GITR during chronic viral infection with LCMV induced early activation and enhanced function of CD4^+^ and CD8^+^ T cells but also preserved virus-specific CD8^+^ T cells from exhaustion (202). In addition, bifunctional macromolecule activating both OX40 and interferon-α signalling displays potent therapeutic effects in mouse HBV models (207).

In addition to therapeutic antibodies, small molecules binding to PD-L1 and driving dimerization and internalization of the ligand have been developed (208). Antigen-specific T and B cell responses from patients with chronic hepatitis B infection were significantly elevated upon PD-L1 small molecule inhibitor treatment. Another strategy is the use of PD-L1 antisense oligonucleotides to degrade PD-L1 transcripts specifically in hepatocytes.

### 4.2 Clinical trials currently evaluating immune checkpoint blockade in HBV patients

Ongoing clinical trials focus mostly on the PD-1/PD-L1/2 pathway by using checkpoint blockade alone or in combination with antiviral agents or vaccines, as resumed in **Table 3**. Although targeting other inhibitory ICs or stimulatory ICs with agonists has been already experienced in clinical trials for cancer immunotherapy (209), it has not yet been launched for CHB patients.

**Table 3:** Ongoing clinical trials in CHB patients involving checkpoint inhibitors.

Clinical trial identifier	Phase	Treatment targeting IC	Combination with other treatments	Number of participants
NCT04133259	2	PD-1 inhibitor (mAb)	NUC (Entecavir or Tenofovir disoproxil fumarate)	44
NCT04778904	1/2	PD-1 inhibitor (mAb)	Therapeutic HBV vaccines	52
NCT04046107	1/2	PD-1 inhibitor (mAb)	None	30
NCT04225715	2	PD-L1 inhibitor (locked nucleic acid (LNA))	siRNA (HBsAg mRNA silencer) and NUC	275 in total, including other treatment arms
NCT04294498	2	PD-L1 inhibitor (mAb)	NUC (Entecavir)	43*
NCT05275023	2	PD-1 inhibitor (mAb)	siRNA (HBV mRNAs silencer) and NUC (Tenofovir Disoproxil or Tenofovir Alafenamide or Entecavir)	44
NCT05242445	1	PD-1 inhibitor (mAb)	None	40
NCT05343481	2	PD-1 inhibitor (mAb)	Therapeutic HBV vaccines	120
NCT04465890	2	PD-L1	None	208
NCT04680598	Prospective Observational Study	PD-1 and PD-L1 inhibitors	Tenofovir or entecavir	800*

*Patients with HBV-related HCC.

A recent phase I study in virally suppressed chronic HBV patients evaluated PD-1 blockade using nivolumab combined or not to HBV vaccine (210). Such treatment was safe and effective for the treatment of virally suppressed patients with CHB, as it led to HBsAg decline in most patients and sustained HBsAg loss in one patient. Many clinical trials based on IC blockade alone or in combination with antivirals or vaccines are currently ongoing in patients with chronic HBV (**Table 3**). Further developments of IC blockers for chronic HBV infection will include different dosages, combination therapies but also careful evaluation for risk of adverse effects and safety profile.

### 4.3 Risk of side effects/rupture of liver tolerance/exacerbation hepatitis

IC blockade present a major risk of affecting hepatic immune tolerance and exacerbation hepatitis, as already experienced in animal models. TIGIT blocking in HBsAg transgenic mice, whose adaptive immune system is tolerant to HBsAg, drives inflammation, chronic hepatitis and promotes HCC, along with the emergence of functional HBsAg-specific CTLs (141). This suggest that TIGIT blockade may brake immune tolerance and favour evolution towards HCC. 4-1BB agonist stimulation exacerbated liver inflammation, leading to fibrosis, cirrhosis and ultimately HCC in HBV transgenic mice, and this effect was mainly mediated by CD8^+^ T cells (151).

## 5 Conclusion and further challenges

Dysregulation of ICs’ expression on T and NK cells is evident in CHB patients. It remains unclear whether the HBV-induced modulation of ICs’ profiles contributes to the establishment of chronic infection by attenuating effector cell responses, as part of an immune escape mechanism induced and triggered by the virus, or whether it is more a protective mechanism engaged by the host immune system to prevent excessive tissue injury. ICs appear to be at the centre of the pathogenesis of HBV infection, and own promising potential for clinical translations as targets for immunotherapies and biomarkers of clinical evolution or response to treatment.

Most of the studies focused on individual ICs, whereas the fate of the T/NK cells will result from the global pattern of ICs expression and co-expression, as well as the expression of corresponding ligands within cells from the liver microenvironment. Therefore, it is crucial to further characterize deeply the global patterns of ICs and IC-ligands expression within the liver microenvironment, as well as ICs in link with the function of the cells and their metabolism. It will allow evaluating the hierarchy of ICs expressed on T and NK cells, their relative role in driving T and NK dysfunction, and guidance for therapeutic developments. Understanding the regulation of ICs’ expression during HBV infection and during the course of antiviral therapies is crucial to bring insights into the pathogenesis of chronic HBV infection and identifying new targets for the development of effective immune-based antiviral therapies in order to propose the best treatment combination. The main challenge for the investigation of ICs’ expression in CHB patients is the limited access to liver tissue of patients, notably NUC-treated patients. Therefore, the modulation of ICs by NUCs has been studied mainly in the blood, and only limited data are available on the intrahepatic level. In summary, NUC therapies seem to normalize the IC landscape of treated patients, demonstrating a reversible phenomenon linked to the arrest of HBV replication and decreased local inflammation.

Soluble ICs impact on the immune system in the context of CHB seems quite complex. Soluble ICs likely perform dual roles in the regulation of immune responses according to the specific type of IC. Therefore, there is still a need to shed light on the contribution of ICs to the disease, and evaluate their potential as prognostic biomarkers of clinical evolution and/or therapeutic response to treatments.

Finally, immunotherapies aimed at blocking inhibitory ICs or boosting stimulatory ones seem promising, and the non-redundant character of inhibitory ICs provides a rationale to target several pathways in combination for optimal responses. However, the restoration of a potent immune response will more likely rely on combinatorial strategies with direct antivirals to lower the viral load and antigen supply, and on immunotherapy to reinvigorate the host immune system including multiple IC blockade, cytokines and therapeutic vaccination. Ongoing clinical trials for CHB patients mainly use this approach – combining antiviral agents with PD-1 or PD-L1 inhibitors. The future research will hopefully show whether this therapeutic strategy will provide an additional boost to maximize the host immune response and bring HBV functional cure closer to reality. Of course, the challenge is to reinvigorate effector cell functions without causing adverse effects due to excessive immune-mediated damage of the liver and rupture of tolerance.

To conclude, functional antiviral T cells and NK cells are required for HBV control. Reinvigorate the dysfunction of these cells represents a promising approach to achieving a functional cure for HBV infection. The future of HBV cure might necessitate a combination of replication inhibition, antigen reduction and immune stimulation (198).

## Author contributions

LD, CA, and ZM contributed to conception, organized the database, and design of the manuscript. LD wrote the first draft and design figures. CA wrote several sections of the manuscript. PM acquired the funding and provided critical feedback. ZM supervised the manuscript and figure preparation and prepared the final version. All authors contributed to the article and approved the submitted version.

## Glossary

**Table:**

4-1BBL	4-1BB ligand
α-syn	α-synuclein fibrils
ALT	alanine aminotransferase
APC	antigen presenting cell
AST	aspartate aminotransferase
BTLA	B and T lymphocyte attenuator
CAR	chimeric antigen receptor
cccDNA	covalently closed circular DNA
CEACAM1	carcinoembryonic antigen-related cell adhesion molecule 1
CHB	chronic hepatitis B
CTL	cytotoxic T lymphocytes
CTLA-4	cytotoxic T lymphocyte antigen-4
DC	dendritic cell
FGL1	fibrinogen-like protein1
Gal-3	galectin-3
Gal-9	galectin-9
GITR	glucocorticoid-induced tumor necrosis factor-related protein
GITRL	GITR ligand
HBeAg	hepatitis B e-antigen
HBsAg	hepatitis B surface antigen
HBV	hepatitis B virus
HCC	hepatocellular carcinoma
HMGB1	high mobility group protein B1
HVEM	herpes virus entry mediator
IC	immune checkpoint
ICOS	inducible T-cell co-stimulator
ICOSL	ICOS-ligand
IFN	interferon
IL	interleukin
LAG-3	lymphocyte activation gene-3
LSEC	liver sinusoidal endothelial cells
LSECtin	liver and lymph node sinusoidal endothelial cell C-type lectin
MHC	major histocompatibility complex
NK	natural killer
NUC	nucleoside/nucleotide analogs
OX40L	OX40-ligand
PBMC	peripheral blood mononuclear cells
PD-1	programmed death protein 1
PD-L1/2	programmed death-ligand 1/2
PS	phosphatidylserine
s4-1BB	Soluble 4-1BB
sCTLA-4	soluble CTLA-4
sGITR	soluble GITR
sIC	soluble IC
sICOS	soluble ICOS
sLAG-3	soluble LAG-3
SLAM	signaling lymphocyte activation molecule
sPD-1	soluble PD-1
sPD-L1	soluble programmed death-ligand 1
sTIM-3	soluble TIM-3
TCR	T cell receptor
Th	T helper
TIGIT	T cell immunoreceptor with immunoglobulin and ITIM domain
TIM-3	T cell immunoglobulin domain and mucin domain 3
TNFR	tumor necrosis factor receptor
Treg	regulatory T cell
WHV	woodchuck hepatitis virus.
